# 5-HT1a Receptor Involvement in Temporal Memory and the Response to Temporal Ambiguity

**DOI:** 10.3389/fnins.2018.00439

**Published:** 2018-07-03

**Authors:** Zvi R. Shapiro, Samantha Cerasiello, Loryn Hartshorne, Matthew S. Matell

**Affiliations:** Department of Psychological and Brain Sciences, Villanova University, Villanova, PA, United States

**Keywords:** 5HT1AR, interval timing, memory, rats, WAY100635, 8-OH-DPAT, stimulus compounding, drug discrimination

## Abstract

It has previously been demonstrated that rats trained on the peak-interval procedure to associate two different cues with two different fixed interval schedules will generate a scalar peak function at an intermediate time when presented with the compound cue. This response pattern has been interpreted as resulting from the simultaneous retrieval of different temporal memories, and a consequential averaging process to resolve the ambiguity. In the present set of studies, we investigated the role that serotonin 1a receptors play in this process. In Experiment 1, rats were trained on a peak-interval procedure to associate the interoceptive states induced by saline and the 5-HT1a agonist, 8-OH-DPAT, with a 5 s or 20 s fixed-interval schedule signaled by the same tone cue (counter-balanced). While peak functions following administration of saline were centered at the appropriate time (5 s or 20 s), peak functions following administration of the agonist were centered around 7 s, irrespective of the reinforced time during training, suggesting agonist-induced disruption in selective temporal memory retrieval, resulting in increased ambiguity regarding the appropriate time at which to respond. In Experiment 2, rats were trained in a peak-interval procedure to associate a tone cue with a 10 s fixed interval and a light cue with a 20 s fixed interval. Administration of the 5-HT1a antagonist, WAY-100635, had no impact on timing when single cues were presented, but altered the intermediate, scalar, response to the stimulus compound, suggesting antagonist-induced disruption in the processes used to deal with temporal memory ambiguity. Together, these data suggest that manipulations of 5HT transmission at the 5-HT1a receptor cause changes in the temporal pattern of responding that are consistent with alterations in temporal memory processes and responses to temporal ambiguity.

## Introduction

Adapting behavior to the temporal regularities in the environment is critical for the efficient utilization of resources. This capacity requires organisms to perceive the duration between events and outcomes, store these durations in memory, and temporally control their behavior using these memories when re-exposed to the predictive events. The ability to perceive and behave with respect to time in the seconds to minutes range, interval timing, has frequently been studied using reproduction procedures, such as the peak-interval procedure (Roberts, 1981). In this procedure, subjects are initially trained on a fixed-interval schedule that a discriminative stimulus (e.g., a tone) signals that reinforcement can be earned for an operant response (e.g., a lever press) once a fixed interval (e.g., 10 s) has elapsed. As there are no external signals specifying when the fixed interval has elapsed and reinforcement can be earned, subjects have to utilize an internally generated estimate of duration to judge when to respond. On non-reinforced probe trials, the signal stays on for several times the fixed-interval duration before terminating in a response-independent manner. Plotting the average response rate as a function of time results in a peak-shaped response function, with the mode (peak time) close to the programmed fixed interval. The peak time indicates the subject’s accuracy in perceiving and acting at the time at which reinforcement is expected. The width of the response function (peak spread) indicates the subject’s temporal precision and/or confidence in this estimate. The coefficient of variation (CV), the peak spread divided by the peak time, is constant across a range of durations, a phenomenon known as the scalar property, demonstrating Weber’s law for temporal perception (Gibbon, 1977).

The vast majority of peak-interval timing studies have utilized test trials in which the duration to be timed is explicitly indicated to the subject. In other words, there is a “correct” response time and location to maximize the likelihood of reinforcement. Considerably less work has examined how subjects deal with ambiguity regarding the appropriate response time. Such ambiguity can occur when animals are trained to time multiple durations. For example, in the “bi-peak” or “tri-peak” procedure, a single cue is used to indicate the availability of reward following one of two or three randomly selected durations (Matell et al., 2003; Meck et al., 2012), and which duration will be reinforced on a particular trial (if any) is unknown until the shorter of the durations have passed. Typically, because subjects are reinforced for responding on different levers at the different durations, the consequences of this initial ambiguity is minimal, and responding at each lever is often centered at each lever’s associated criterion duration (although, see Leak and Gibbon, 1995; Matell and Meck, 1999; Meck et al., 2012 for alternative outcomes). Ambiguity can also occur if different signals, each previously associated with a different duration, are presented simultaneously (i.e., a compound stimulus). In this case, the “correct” time to respond is not specified, and subjects may attempt to resolve this discrepancy. Understanding how subjects behave in these situations can provide clues to the composition of the interval timing system and the corresponding representation of time.

We, and others, have conducted a number of studies investigating how animals respond to ambiguous temporal information resulting from the presentation of compound stimuli. Under certain circumstances, subjects will behave as though they have computed a weighted average of the component durations, and then timed this average in an otherwise normal manner (Cheng and Roberts, 1991; Swanton et al., 2009; Swanton and Matell, 2011; Kurti et al., 2014; Matell and Kurti, 2014; De Corte and Matell, 2015; Delamater and Nicolas, 2015; Matell et al., 2016). For example, Swanton et al. (2009) trained rats in a two cue, two duration peak-interval procedure that a tone signaled a 10 s fixed interval and a light signaled a 20 s fixed interval. After responding to each component cue was temporally controlled, we tested the rats’ responses to the simultaneous compound (tone + light), presented on non-reinforced probe trials only. Responding on these compound probes was maximal at an intermediate location between the two component peaks. Importantly, the response to the stimulus compound was peak-shaped, normal, and scalar. Specifically, the peak time and peak spread of the compound peak function were midway between those of the 10 s and the 20 s component peak functions. These results suggest that the simultaneous presentation of the two component cues resulted in retrieval of both the 10 s and 20 s temporal memories. Because these two temporal memories were discrepant, the rats appear to have averaged these temporal memories into a single expectation, which they then timed in an otherwise normal manner. We also examined the response of the counter-balanced modality-duration pairing (i.e., light = 10 s, tone = 20 s). While the same scalar intermediate response pattern was seen during the first block of testing, there was a trend for the response pattern to become asymmetric with a bias toward the light with subsequent testing. As such, it appears that rats can utilize different behavioral repertoires when faced with discrepant or competing temporal information (see below).

A number of subsequent studies have confirmed and extended these basic findings. Swanton and Matell (2011) and Kurti et al. (2014) examined the response pattern to compound cue presentation using a range of component durations with broader ratios than used in Swanton et al. (2009), (i.e., 4 s/12 s, 8 s/24 s, 5 s/20 s, and 5 s/30 s). In all cases, we found scalar temporal responding at an intermediate duration to the compound cue (i.e., temporal averaging) when the tone signaled the short duration and the light signaled the long duration. Further, we found that the time of the compound peak was well predicted by a weighted geometric average of the component durations with the relative reinforcement probabilities of each cue serving as weights. In contrast, we found non-scalar, rightward-skewed, response patterns when the light signaled the short duration and the tone signaled the long duration. When the durations were maximally discrepant (5 s/30 s), light-short/tone-long rats showed a bimodal response function.

Matell and Kurti (2014) differentially varied the reinforcement probabilities of the two cues so that the short or long cue was more valuable than the other cue. Under light-short/tone-long conditions, increasing the value of one of the cues over the other resulted in a selection-like response pattern, such that the compound peak function largely overlapped whichever cue was more valuable. Under tone-short/light-long conditions, the position of the scalar compound peak moved toward the more valuable cue. Intriguingly, when the short cue was substantially more valuable than the long cue (i.e., when the reinforcement rate was 6:1), the compound peak was asymmetrical and rightward-skewed in a manner similar to the light-short/tone-long rats seen previously. These data further indicate that rats can utilize different strategies when dealing with discrepancy or ambiguity in signaled temporal expectations.

De Corte and Matell (2015) further examined this apparent flexibility in compound responding by training rats on a 5 s/20 s peak procedure with tones and lights signifying the two durations (counter-balanced). However, the response manipulandum associated with each duration was spatially segregated (e.g., tone signaled a 5 s fixed interval schedule requiring a response on the left nosepoke, light signaled a 20 s fixed interval schedule requiring a response on the right nosepoke). When tested with the compound cue, all rats showed three different response patterns, which varied across trials. In tone-short/light-long rats, the most common pattern (75% of trials) was to respond solely on the light-associated long nosepoke, with a peak that was leftward shifted from the light-alone peak (temporal averaging favoring the light). In the second most common pattern (13% of trials), they responded solely on the tone-associated short nosepoke with a peak that was rightward shifted from the tone-alone peak (temporal averaging favoring the tone). In light-short/tone-long rats, the most common pattern (60% of trials) was to respond solely on the light-associated short nosepoke in a veridical manner (selection responding). The second most common pattern was a bimodal response, with both peaks veridical (25% of trials – bimodal without bias). However, on 15% of trials, the rats responded on the tone-associated long nosepoke, with a peak that was leftward shifted from the tone alone peak (temporal averaging favoring the tone). These results again demonstrate that a variety of response strategies or patterns can be generated under conditions of ambiguity, and that even when rats utilize an integration or averaging strategy, the bias or weighting given to the different temporal memories is flexible.

We have also demonstrated that rats can be trained in the peak-interval procedure to use interoceptive cues to predict the time of reinforcement. Using a drug-discrimination approach, in which the administration, and subsequent interoceptive effects, of a pharmacological agent serve as the discriminative stimulus for operant responding (Kubena and Barry, 1969; Krimmer et al., 1984; McMillan and Hardwick, 2000; McMillan and Li, 2000), Kurti et al. (2014) trained rats that when saline was administered before the experimental session, a tone cue signaled that reinforcement was probabilistically available after 5 s. However, if a low dose of amphetamine (0.5 mg/kg) was administered prior to the experimental session, the same tone cue signaled that reinforcement was probabilistically available after 20 s. An examination of data from the first five trials of each session (before feedback) demonstrated that the rats could accurately use their interoceptive state to predict the time of reinforcement, as they peaked at 5 s following saline injections, but they peaked at 20 s after receiving amphetamine. Similar results were obtained when rats were given saline or amphetamine injections immediately before final test sessions composed entirely of probe trials. Remarkably, if an intermediate dose of amphetamine was given prior to one of these test sessions, the rats peaked at an intermediate location in a scalar manner. These data were interpreted as indicating that an intermediate dose led to an interoceptive state that was ambiguous with regard to the appropriate temporal memory to utilize. In a manner similar to that seen when external cues are presented in compound, the rats seemed to deal with this ambiguity by responding in a scalar manner at an average of the trained durations.

A classic finding in the interval timing literature is that dopaminergic psychostimulants, such as amphetamine, methamphetamine, and cocaine can increase the speed of an internal clock (Maricq et al., 1981; Maricq and Church, 1983; Meck, 1983; Buhusi and Meck, 2002; Matell et al., 2004, 2006). For example, Meck (1996) trained rats on a single duration peak procedure before giving them a methamphetamine (or saline) injection immediately prior to test sessions. He found that their peak functions on methamphetamine were shifted leftward (i.e., earlier in time) compared to their peak functions on saline, which were not different from their training peak functions. Importantly, the size of the leftward shift was proportional to the duration being timed, thereby suggesting that methamphetamine speeds up the clock. It is for this reason that Kurti et al. (2014) elected to train the rats that amphetamine was associated with the long, rather than short, duration. If amphetamine had been associated with the short duration, the argument could be made that a short peak time following amphetamine was (at least partially) due to an increase in clock speed rather than identification of a drug-induced interoceptive state as a timing cue. However, because we trained the rats that amphetamine was associated with a longer duration, any clock speed effects would have weakened the effect, rather than contributed to it.

Although Kurti et al. (2014) designed their experiment to negate a clock-speed interpretation, this relationship may be a lingering concern for some. As serotonin has minimal effects on clock speed (see Ho et al., 2002 for review), we sought to investigate whether a 5-HT-induced interoceptive state could be used as a signal for the delay to reinforcement. We conducted a similar study to Kurti et al. (2014), but here using the 5-HT1a agonist, 8-OH-DPAT, as the drug. Unexpectedly, and in contrast to the results obtained with amphetamine, while administration of saline led to peak responding at the appropriate time, administration of 8-OH-DPAT did not lead to responding centered at the duration associated with this drug state. Rather, responding peaked to the right of the shorter duration, suggesting that stimulation of the 5-HT1a receptor disrupts selective temporal memory retrieval and promotes memory integration. To further explore this possibility, we conducted a second, follow-up, experiment to evaluate whether the 5-HT1a antagonist, WAY-100635, would disrupt temporal averaging. In Experiment 2, we trained rats to associate tones and lights with two different durations using contingencies found to promote temporal averaging when tested with an ambiguous compound cue (Swanton et al., 2009). Rats given saline peaked, in a scalar manner, at an intermediate duration when presented with the compound cue. In contrast, and consistent with our interpretation of Experiment 1, the time of responding on compound trials became more similar to those for the short or long duration cue when testing followed WAY-100635 administration.

## Experiment 1

### Method

#### Subjects and Apparatus

Twenty male Sprague-Dawley rats (Harlan), approximately 90 days of age at the onset of the experiment, were subjects. The animals were housed two per cage and given *ad libitum* access to food and water until the experiment began. One week prior to training, food access was restricted and the rats were maintained for the remainder of the experiment at approximately 85–90% of their free-feed weight, adjusted for growth. Prior to beginning training, approximately ten 45 mg grain pellets (Formula F; Noyes Precision, Lancaster, NH, United States) were given to each rat in their home cage to acclimate them to the reinforcer used in the operant chambers. All animals were kept on a 12-h light:dark cycle (lights on at 8:00 AM – rats were trained and tested during the light cycle). Food rations were given shortly after each daily session. Unless noted otherwise, all training and testing sessions lasted 120 min. All procedures were approved by the Institutional Animal Care and Use Committee of Villanova University.

Training and testing took place in standard operant conditioning chambers described in detail previously (Swanton and Matell, 2011). Three nosepoke apertures (2.5 cm diameter) lined the back wall, equidistant from each other. Only the center nosepoke was used in these experiments. A nosepoke response was recorded each time the rat inserted its snout in the nosepoke aperture (thereby breaking an infrared photobeam), and it was required to remove its snout from the nosepoke before another response could be recorded. This arrangement (reinforcing snout insertion rather than nosepoke aperture occupancy) has been found to generate temporally controlled behavior that is similar to that seen with lever presses as the operant (Matell et al., 2006; Gooch et al., 2007). A 95 dB 1 kHz tone served as the discriminative stimulus and 45 mg grain pellets provided reinforcement. A fan in the chamber provided ventilation, and provided a 60 dB background sound level. Responses were recorded and stimuli controlled using Graphic State 3 software with a 20 ms resolution (Coulbourn Instruments, Whitehall, PA, United States).

#### Drugs

8-OH-DPAT was dissolved in 0.9% physiological saline (250 μg/ml), and either the drug or vehicle (0.9% saline) was administered subcutaneously just prior to each training and test session at a volume of 1.0 ml/kg. Drug and vehicle injections were counterbalanced across days in 2-day blocks, with the order in each block randomized.

#### Procedure

Rats were given 12 sessions of fixed-interval training in which the first nosepoke response made either 5 s (short) or 20 s (long) following onset of a 1 kHz auditory cue was reinforced with a 45 mg grain pellet, at which point the cue terminated, ending the trial. Responses made prior to the completion of the fixed interval had no programmed consequence. The FI duration in place for each session depended on whether the rat was given saline or the 5-HT1a agonist immediately beforehand. In group Saline-short/Drug-long, saline was always paired with the short 5 s FI and 8-OH-DPAT was always paired with the long 20 s FI. In group Drug-short/Saline-long, 8-OH-DPAT was always paired with the short 5 s FI and saline was always paired with a 20 s FI. Rats in group Saline-short/Drug-long were trained and tested during the spring, and rats in group Drug-short/Saline-long were trained and tested during the summer. Trials were separated by a uniform 60–80 s inter-trial interval.

Following these 12 FI sessions, rats were given 16 sessions of peak-interval training, with the same drug-duration association. The first five trials of each session were non-reinforced probe trials, and the remaining trials were composed of 75% FI trials, and 25% probe trials, resulting in means of 16.7 (1.5) and 16.2 (1.4) probe trials per session on saline and drug days, respectively. Although data from FI trials were not saved, the expected mean number of FI trials per session would be 34.3. Probe trials lasted 60–80 s and terminated independently of responding. We ran the initial non-reinforced probe trials to allow assessment of temporal expectation associated with the saline/drug injection, prior to any feedback about the correct duration for that day.

Following these PI sessions, the rats were given three test sessions in which all trials were non-reinforced probes, and no feedback was provided, resulting in a mean of 51.4 (0.5) probe trials per session. Prior to each of these test sessions, the rats were given an injection of saline, the training dose of the 5-HT1a agonist (250 μg/kg), or an intermediate dose of the 5-HT1a agonist (125 μg/kg), with the order of administration counter-balanced across rats. In between each of these test sessions, a saline PI training session and a drug PI training session were conducted.

#### Analysis

Nosepoke entries were binned into 1-s bins to create peak-response functions. Data from the last eight PI training sessions (four drug and four vehicle) were pooled for analysis. Data from the test sessions (all probe trials) were analyzed separately. Due to the skewed pattern of responding frequently seen following drug administration (see Results), rather than use a standard Gaussian to describe the data, we fit (curve fitting package of MATLAB, Cambridge, MA, United States) the pooled responses with a dual asymmetric sigmoid function, Y = Y_0_ + A ^∗^ (1/(1 + exp(-1^∗^((x - B + C/2)/D)))) ^∗^ (1-(1/(1 + exp(-1^∗^((x - B - C/2)/E))))), as used previously (Swanton and Matell, 2011; Matell and Kurti, 2014). Y_0_ is the baseline, A is a scaling factor, peak time was taken as B, peak spread was taken as C, while D and E are parameters that contribute to the shape of each sigmoidal half. The 5-parameter, asymmetric peak function did an excellent job fitting the data, with mean *R*^2^ values of 0.968 (0.043) for the saline peaks, and 0.934 (0.0856) for the drug peaks. The CV was computed by dividing peak spread by peak time.

Single trial analyses were conducted by fitting the response pattern on individual trials with a series of three flat lines to capture the initial low response rate, a high response rate, and a terminal low response rate (Church et al., 1994). Start and stop times were taken as the time bins at which the high response rate line begins and ends. To minimize the influence of temporally uncontrolled responding that can bias the transition statistics (Matell et al., 2006), we constrained the analyses such that the start time was required to occur earlier than twice the obtained peak time for that subject, and the stop time was required to occur later than half the obtained peak time for that subject as done previously (De Corte and Matell, 2015). Statistics are reported as mean (standard deviation).

Because the two groups were trained and tested in different months (spring and summer), repeated measure analysis of variance (ANOVA) were conducted for each group separately using SPSS (v24), with an alpha level set at 0.05. LSD *post hoc* tests were conducted when relevant. The current session’s drug state (Drug) served as the within subject factor.

### Results

#### PI Sessions

**FIGURE 1:**
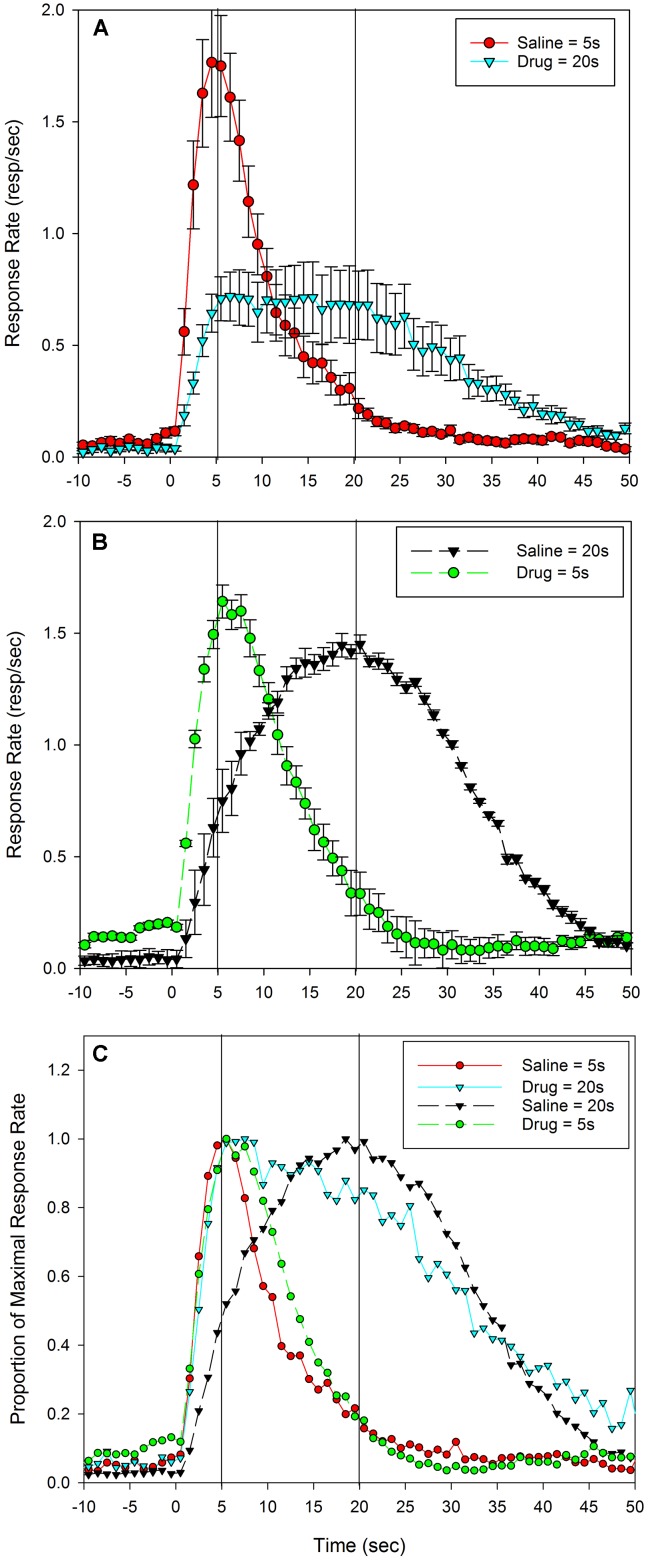
Mean peak response functions during probe trials from training sessions in which an injection of vehicle (saline) or drug (5-HT1a agonist, 8-OH-DPAT) was given prior to each session, and the fixed-interval in place on that session depended on the substance administered. In half the rats **(A)**, Saline = short (5 s) and 8-OH-DPAT = long (20 s), for the other half of the rats **(B)**, this relationship was reversed, 8-OH-DPAT = short (5 s) and Saline = long (20 s). **(A,B)** Indicate standard errors. **(C)** The same data after normalizing by maximal response rate, to allow easier comparisons of the time of responding irrespective of changes in response rate. It also plots both groups together to allow for easier comparisons between groups. In **(A–C)**, vertical lines at 5 s and 20 s indicate programmed times of reinforcement availability.

The response rates on drug days in the Saline-short/Drug-long group during the PI training sessions were extremely low, preventing an assessment of temporal control prior to the rats receiving feedback. Therefore, the data from probe trials following these initial five trials were analyzed. Response rates as a function of time on these PI sessions are plotted in **Figure 1**, separately for the two groups and split by administered drug; group Saline-short/Drug-long’s peak functions are shown **Figure 1A** and group Drug-short/Saline-long’s peak functions are shown in **Figure 1B**. **Figure 1C** depicts these same data as a proportion of maximal response rate over time to allow easier comparison of the timing of responses irrespective of changes in response rate, and to allow comparison across groups.

As can be seen, following administration of saline, rats peaked at a time that corresponded to their condition; rats in group Saline-short/Drug-long (**Figures 1A,C** – solid red line) peaked around the short peak time of 5 s, whereas rats in group Drug-short/Saline-long peaked around the long peak time of 20 s (**Figures 1B,C** – dashed black line). In contrast, following administration of 8-OH-DPAT, rats appeared to peak later than 5 s in the Drug-short/Saline-long group (**Figures 1B,C** – dashed green line), and peak earlier than 20 s in the Saline-short/Drug-long group (**Figures 1A,C** – solid cyan line). Specifically, the mean peak time following saline administration in group Saline-short/Drug-long was 4.6 s (0.9) and the mean peak time following saline administration in group Drug-short/Saline-long was 16.8 s (2.5). The mean peak time following 8-OH-DPAT administration in group Saline-short/Drug-long was 7.6 (3.5) and the mean peak time following 8-OH-DPAT in group Drug-short/Saline-long was 5.8 s (1.4).

Repeated measure ANOVAs conducted on peak times with Drug as a within-subject factor demonstrated significant effects for both groups [Saline-short/Drug-long: *F*(1,9) = 5.69, *p*< 0.05; Drug-short/Saline-long: *F*(1,9) = 168.47, *p*< 0.001].

The CV (peak spread normalized by peak time) in group Saline-short/Drug-long was 1.95 (0.61) following saline and 4.08 (2.42) following 8-OH-DPAT. In group Drug-short/Saline-long, the CV was 1.81 (0.34) following 8-OH-DPAT, and 1.74 (0.41) following saline. A repeated measures ANOVA on relative spreads revealed significant effects of Drug in the Saline-short/Drug-long group [*F*(1,9) = 6.647, *p*< 0.05], but not in the Drug-short/Saline-long group [*F*(1,9) < 1]. These differences in CV in the Saline-short/Drug-long group are likely due to changes in response times over the session due to relearning, as they were not seen during the test sessions (see below).

We also assessed whether poke duration (i.e., the time from the entry of the snout in the nosepoke aperture to its exit) changed as a result of drug administration. Mean poke durations for each subject were 0.49 s (0.15) and 0.51 s (0.10) for the short and long associated durations, respectively, in the Saline-short/Drug-long group. For the Drug-short/Saline-long group, poke durations were 0.29 s (0.06) and 0.32 s (0.09) for the short and long associated interoceptive cues, respectively. Repeated measure ANOVAs revealed no differences in either group [Saline-short/Drug-long: *F*(1,9) < 1; Drug-short/Saline-long: *F*(1,9) = 4.08, *p* = 0.074].

These data indicate that administration of the 5-HT1a agonist produced a large leftward shift, and a broadening of the response function, in rats trained to associate the interoceptive drug state with a reinforcement time of 20 s, but a small rightward shift in the rats trained to associate the interoceptive drug state with a reinforcement time of 5 s. The opposite direction of these results is consistent with a 5-HT1a agonist induced disruption in temporal memory encoding and/or retrieval processes for the temporal memory associated with the rats’ interoceptive drug state. However, these data likely underestimate the impact of the 5-HT1a agonist as the rats were given feedback regarding the appropriate reinforced duration during the session (in the form of reinforced fixed-interval trials). As such, the need to retrieve the appropriate reference memory given the current drug state was dramatically diminished. Therefore, we subsequently tested the rats by conducting three test sessions in which only probe trials were presented, thereby preventing any feedback which might facilitate utilization of the appropriate temporal memory.

#### Test Sessions

**FIGURE 2:**
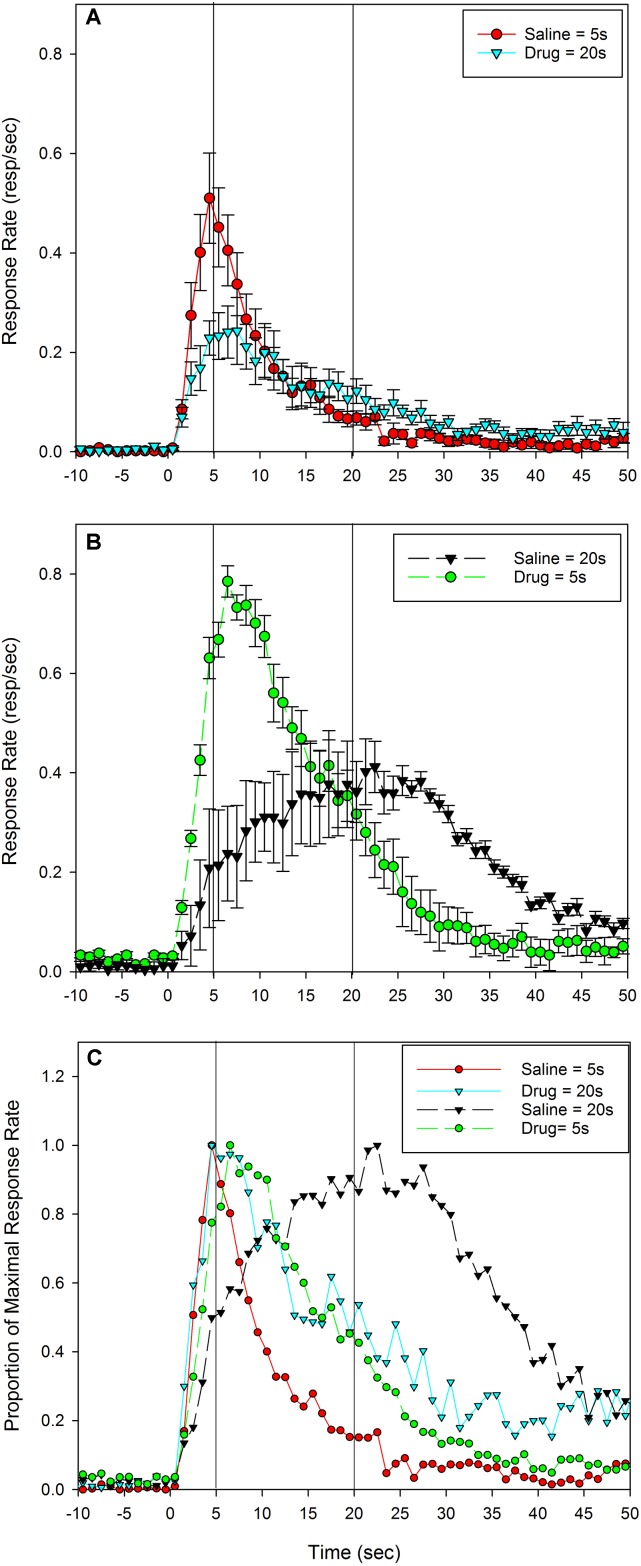
Mean peak response functions from probe trials during the testing sessions following an injection of Saline or 8-OH-DPAT. No reinforcement was provided during these sessions, so responding was controlled solely by expectations resulting from subjects’ interoceptive state. Panel **(A)** shows rats in which Saline = short (5 s) and 8-OH-DPAT = long (20 s), and panel **(B)** shows rats in which this relationship was reversed, 8-OH-DPAT = short (5 s) and Saline = long (20 s). Panel **(C)** plots the both groups together after normalizing by maximal response rate, to allow easier comparisons across groups of the time of responding irrespective of changes in response rate. All figure details are equivalent to **Figure 1**.

Response rates as a function of time since cue onset during the test sessions are plotted in **Figure 2A** (Saline-short/Drug-long) and **Figure 2B** (Drug-short/Saline-long), split by administered drug.

To allow better comparison of the time of responding across conditions, these same data are plotted together and as a proportion of maximal response rate in **Figure 2C**. As can be seen, the data are similar to the training session data presented in **Figure 1**, with two important exceptions. First, the peak response function following 8-OH-DPAT in the Saline-short/Drug-long group (**Figures 2A,C**; solid cyan line) peaks were earlier and had greater rightward (positive) skew than it did in the training sessions. Similarly, the peak response function following 8-OH-DPAT in the Drug-short/Saline-long group (**Figures 2B,C**; dashed green line) was later than it was in the training sessions. As a consequence, the peak functions following drug administration in both groups were surprisingly overlapping during these test sessions (**Figure 2C**). Specifically, the mean peak times in the Saline-short/Drug-long group were 4.6 s (0.8) following saline administration and 6.3 s (2.4) following 8-OH-DPAT administration. For the Drug-short/Saline-long group, the mean peak times were 19.1 s (7.1) following saline administration and 7.5 s (2.5) following 8-OH-DPAT administration.

These apparent differences in peak times were confirmed with a repeated measures ANOVA, with Drug as a within-subject factor. There was a main effect of Drug in both groups [Saline-short/Drug-long: *F*(1,9) = 6.84, *p*< 0.05; Drug-short/Saline-long: *F*(1,9) = 32.27, *p*< 0.001]. Further, one sample *t*-tests demonstrated that the peak times on saline were not different from the programmed criterion duration of 5 s [Saline-short: *t*(9) = 1.86] and 20 s [Saline-long: *t*(9) = 0.39]. In contrast, the peak times on drug were different from their respective programmed criterion durations [Drug-long: *t*(9) = 18.22, *p*< 0.001; Drug-short: *t*(9) = 3.07, *p*< 0.05].

This phenomenon, that the rats could discern the two drugs states and retrieve the appropriate corresponding duration when the drug signaled the short duration, but would not be able to respond at the appropriate duration when the drug signaled the long duration, coupled with the remarkable similarity in the peak functions following 8-OH-DPAT administration across the groups, is similar to some of the partial or incomplete averaging phenomena seen before (Swanton and Matell, 2011; Matell and Kurti, 2014). Therefore, we also assessed whether drug-associated peak times differed across groups. A one-factor ANOVA comparing the drug-associated peak times across groups provided no support for a difference in response times [*F*(1,18) = 1.11]. We note that because the two groups were tested in different months, this between group comparison should be interpreted cautiously.

The CVs were 1.82 (0.86) for the saline data in the Saline-short/Drug-long group, 2.46 (1.59) for the drug data in the Saline-short/Drug-long group, 1.82 (0.67) for the drug data in the Drug-short/Saline-long group, and 1.82 (0.54) for the saline data in the Drug-short/Saline-long group. Repeated measure ANOVAs with Drug as a within-subject factor yielded no significant results [all *F*’s < 1.3].

Rats in the Saline-short/Drug-long group poked for 0.50 s (0.13) following saline administration, and for 0.54 s (0.13) following 8-OH-DPAT administration. Rats in the Drug-short/Saline-long group poked for 0.35 s (0.09) following saline administration and 0.33 s (0.08) following 8-OH-DPAT administration. A repeated measures ANOVA with Drug as the within-subject factor, revealed no significant effects [Saline-short/Drug long: *F*(1,9) = 2.32, *p*> 0.10; Drug-short/Saline long: *F*(1,9) = 2.69, *p*> 0.10].

All rats were also given an intermediate dose of the drug (125 μg/kg) during a non-reinforced probe session in order to assess how rats would respond to an interoceptive state that was presumably similar to both the training drug state and the training saline state. However, given that the full dose of the training drug did not generate peak response functions that were in the appropriate location, interpretation of these sessions is not immediately clear. For completeness, the peak times following the intermediate dose were 7.3 s (2.1) in the Saline-short/Drug-long group, and 8.3 (3.0) in the Drug-short/Saline-long group. Repeated measure ANOVAs with Drug (Saline, Intermediate Dose, Training Dose) as a factor indicated main effects in both groups: [Saline-short/Drug-long: *F*(2,18) = 8.12, *p*< 0.005; Drug-short/Saline-long: *F*(2,18) = 28.54, *p*< 0.001]. *Post hoc* comparisons indicated significant differences between saline and the intermediate dose (*ps* < 0.005) in both groups, but not between the intermediate and full training dose in either group (*p*s > 0.25). The coefficients of variation were 1.75 (1.09) for the Saline-short/Drug-long group, and 2.33 (0.87) for the Drug-short/Saline-long group. No significant differences in relative spreads were found: [Saline-short/Drug-long: *F*(2,18) = 1.25, *p*> 0.10; Drug-short/Saline-long: *F*(2,18) = 2.42, *p*> 0.10].

#### Integration Versus Selection

**FIGURE 3:**
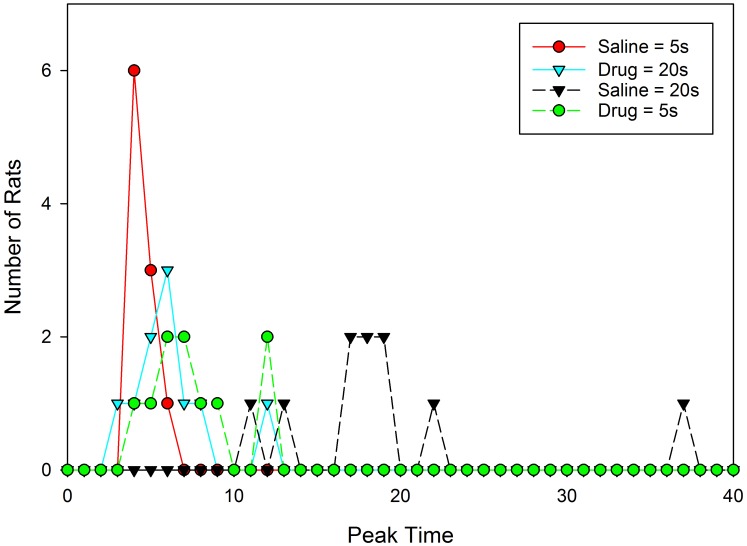
Distribution of individual rats’ peak times from the testing sessions.

Given the skew apparent in the mean peak functions following drug administration, an important question is whether different rats are peaking at different times and/or whether the rats are switching the temporal memories utilized on each trial from one to the other criterion duration, and whether either of these behaviors is more frequent under drug than vehicle. We restrict our analysis here to the data from the test sessions, as no opportunity for relearning is present due to solely presenting probe trials. **Figure 3** shows the distribution of peak times from the test sessions plotted separately for each drug and group. As can be seen, peak times were generally near their associated programmed criterion durations of 5 s or 20 s when under the influence of saline, but fell around 6–7 s when under the influence of 8-OH-DPAT.

We evaluated the difference in relative variation between these distributions (i.e., we accounted for expected scalar variance), by dividing each subjects’ peak time by the mean peak time for that group and drug condition. Neither Mauchly’s test of sphericity, nor Levene’s test for homogeneity of variance, revealed significant effects (Mauchly’s W = 1.0; Levene Statistic = 1.68 and 0.18 for saline and 8-OH-DPAT, respectively). These data suggest that 8-OH-DPAT did not increase relative peak time variation across subjects. Rather, the figure is clear in showing that unlike the saline condition, the peak times in the vast majority of subjects during the drug-conditions are not around the reinforced durations, suggesting that 8-OH-DPAT is disrupting the ability to selectively use the appropriate duration given the drug state.

We also examined whether subjects were utilizing different temporal memories on different trials by conducting single trial analyses, which identify the times on each trial at which there are abrupt transitions in response rates [i.e., from a low rate to a high rate (start time) and from a high rate to a low rate (stop time)]. The mean start and stop times in each condition are presented in **Table 1**.

**Table 1:** Single trial transition times (Experiment 1).

	Saline-short	Drug-long	Drug-short	Saline-long
Start	5.85	7.65	7.42	9.65
*SD*	1.26	1.22	1.27	0.82
Stop	12.52	15.49	16.60	26.09
*SD*	2.32	3.33	2.15	2.95

As with the peak functions, the start and stop times were most similar to the reinforced durations following saline, and were at intermediate locations following administration of 8-OH-DPAT. A repeated measures ANOVA on the Saline-short/Drug-long data with Transition (start, stop) and Drug as within subject factors revealed the expected main effect for Transition [*F*(1,9) = 151.98, *p*< 0.001] as well as an effect of Drug [*F*(1,9) = 9.84, *p*< 0.05], but no interaction [*F*(1,9) = 1.32, *p*> 0.10]. For the Drug-short/Saline-long group, there was the expected main effect of Transition [*F*(1,9) = 517.26, *p*< 0.001], a main effect of Drug [*F*(1,9) = 50.89, *p*< 0.001], as well as a Transition × Drug interaction [*F*(1,9) = 42.40, *p*< 0.001]. *Post hoc* pairwise comparisons indicated significant differences in both start times and stop times across drug states (both *p*< 0.001).

**FIGURE 4:**
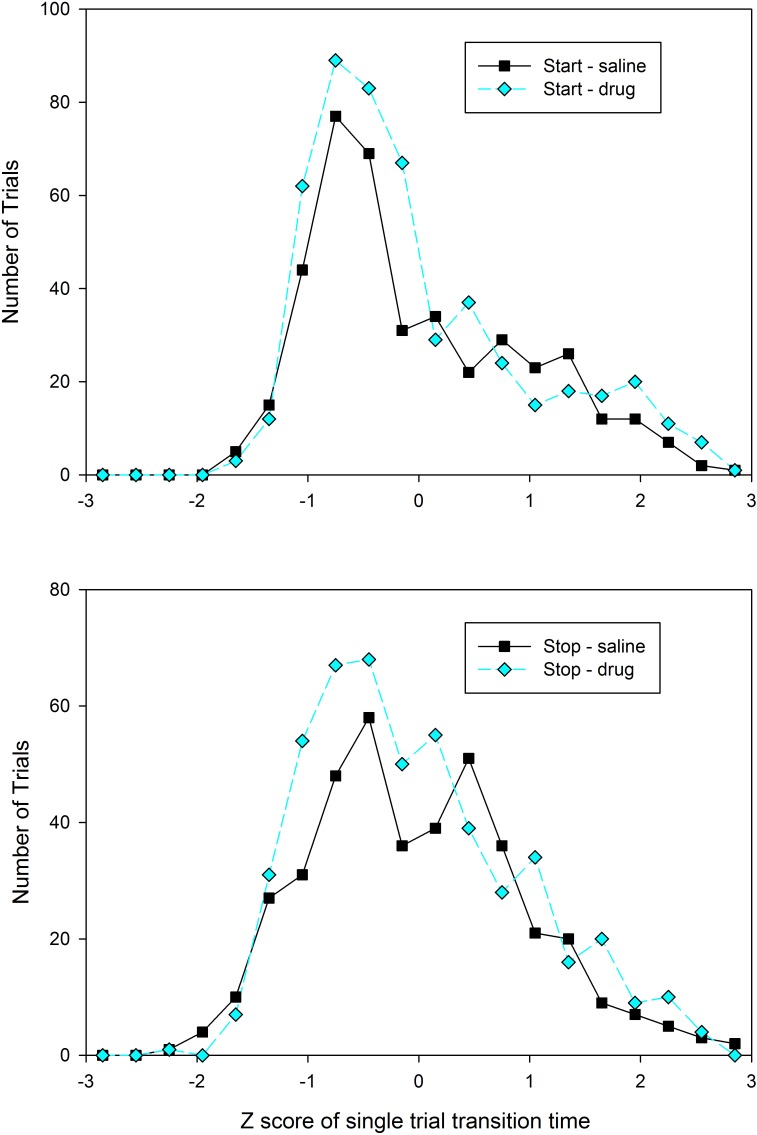
Distribution of the *Z*-scores of each trials’ start **(Top)** and stop **(Bottom)** times from the testing sessions, split by drug condition (black squares = Saline, cyan diamonds = 8-OH-DPAT). *Z*-scores were computed separately for each rat and each condition.

As described above, it is possible that subjects utilized different temporal memories on different trials, particularly following drug administration. Examination of the distribution of start and stop times in individual subjects was not obviously supportive of either a unimodal or bimodal distribution of transition times in any condition or group, presumably due to the low response rates leading to relatively small numbers of trials with identifiable step functions (mean number of trials = 23.7, *SD* = 9.3). Therefore, in order to assess whether the distributions were unimodal or bimodal, we pooled the transition times across subjects. As this pooling would potentially mask bimodality due to between subject variability, we normalized each subject’s distribution by converting the transition times to *z*-scores (individually for each rat). As shown in **Figure 4**, only the stop times under saline administration show any evidence of bimodality in transition times, suggesting that 8-OH-DPAT’s generation of intermediate peak times is not due to an enhancement in the selection of different temporal memories on different trials.

### Discussion

In both the training sessions and the no-feedback test sessions, the peak functions following administration of 8-OH-DPAT were shifted toward an intermediate time, irrespective of the drug-duration relationship in place during training. Specifically, in those rats for whom saline and 8-OH-DPAT were associated with the short and long fixed intervals of 5 s and 20 s, respectively, the peak function following saline administration was located near the 5 s fixed interval, whereas the peak function following 8-OH-DPAT largely overlapped the saline peak function on the left, but with a broad, right tail that extended past 20 s. Conversely, in those rats for whom saline and drug were associated with a long and short fixed interval of 20 s and 5 s, respectively, the peak function following saline was centered around 20 s, whereas the peak function following drug administration was located to the right of the appropriate 5 s time, and again had a broad right tail that extended past 20 s. In fact, the peak functions following drug administration were remarkably similar in the two groups during testing, despite the fact that these groups were reinforced at very different times given their drug state during training sessions. These findings suggest that the administration of the 5-HT1a agonist, 8-OH-DPAT, disrupts selective memory retrieval, resulting in ambiguity regarding the expected time of reinforcement. Examining the distribution of single trial transition times suggests that these intermediate, skewed, peak functions were not the result of different temporal memories being utilized on different trials. Rather, the rats appeared to be influenced by both the short and long temporal expectations, and dealt with this ambiguity by responding at intermediate times. It is, of course, conceivable that the rats cannot discriminate the interoceptive state generated by saline from that induced by 8-OH-DPAT, and this failure to discriminate is the basis for the ambiguity. However, this explanation is difficult to reconcile with our finding that the peak functions fell at the appropriate times following saline administration. If the rats were unable to discriminate their interoceptive states, the saline and drug peak functions should have overlapped. Furthermore, Ybema et al. (1993) demonstrated that rats are able to discriminate 8-OH-DPAT from saline at doses that are both lower (100 μg/kg) and higher (2.5 mg/kg) than those used here. Our experimental design required sufficient discriminability of drug state, which motivated the utilized dose. We note that it is possible that the use of a lower dosage might provide a clearer response pattern, given that 8-OH-DPAT administration produces a dose-dependent reduction in response rate (Dourish et al., 1985).

The present results are reminiscent of the migration effect seen in Parkinson’s patients that were trained and tested off their dopaminergic replacement medication with two typically discriminable durations of 8 s and 21 s (Malapani et al., 1998). Like the present results, these patients’ temporal productions of the short interval were long, while their productions of the long interval were short. A follow-up study (Malapani et al., 2002) revealed that this regression of responding toward the mean of both experienced durations occurred only when patients were tested off their dopamine-replacement medication (irrespective of medication state during training), but not when they were tested on their medication (irrespective of medication state during training). As such, the migration effect was interpreted as resulting from a deficit in memory retrieval, such that both the short and long temporal memories were retrieved, leading to ambiguity regarding the correct response time, and resulting in responding influenced by both durations. While Parkinson’s disease results from a loss of dopaminergic neurons in the substantia nigra pars compacta, disruptions in serotonergic signaling are also seen (Politis and Niccolini, 2015). Indeed, dopaminergic and serotonergic interactions in healthy subjects are well known (see Niederkofler et al., 2015 for review). As such, the current work suggests that the migration effect found in Parkinson’s patients may relate to these serotonergic alterations.

The current results are also similar to the stimulus compounding work described in the introduction in which rats trained that different modality cues are associated with different times of reinforcement (e.g., tone = 5 s, light = 20 s) will exhibit scalar peak responding at an intermediate duration when presented with an ambiguous compound cue (tone + light) (Swanton and Matell, 2011; Kurti et al., 2014). However, the current data do not appear to reflect complete (scalar) temporal memory averaging following drug administration. The rats’ peak functions were rightward (positively) skewed, indicating they initially responded in anticipation of reward around 5 s and then continued responding as though they expected reinforcement at a longer duration. However, the right tail of the drug-associated peaks did not extend as far as the right tail of the 20 s saline peak function, suggesting the rats switched from timing the short duration to timing an integrated expectation of the short and long durations. Intriguingly, a nearly identical response pattern has been seen in our compounding studies when the short cue is more valuable than the long cue (Matell and Kurti, 2014) or when a light signals the short duration, and a tone signals the long duration (Swanton and Matell, 2011), which may reflect intrinsic valuation differences between modalities (Weiss et al., 1993). Together, these data suggest that 5-HT1a receptor agonism impairs the use of an appropriate temporal memory to guide responding, resulting in partial or incomplete averaging like behavior (i.e., responding at an intermediate duration in a skewed manner).

## Experiment 2

Experiment 1 showed that in rats trained that their interoceptive state indicates the appropriate time of reinforcement (either 5 s or 20 s), administration of 8-OH-DPAT led to a response pattern in which responding peaked around 7 s, with a significant rightward (positive) skew. This response pattern is very similar to the “partial” or “incomplete” averaging behavior seen in previous peak-interval compounding studies (Swanton and Matell, 2011; Matell and Kurti, 2014). Our findings suggest that 8-OH-DPAT generates such temporal averaging behavior as a result of drug-induced disruptions to timing processes at retrieval, as seen in the migration effect (Malapani et al., 1998, 2002). If instead, there was a failure to selectively encode the time of reinforcement following 8-OH-DPAT administration, such that the durations stored in memory were not selectively associated with the corresponding interoceptive state, subjects would be expected to show inaccurate responding under both saline and drug. As rats tested on saline showed veridical responding, these data suggest that temporal memories were selectively stored, and that subjects could accurately discriminate their interoceptive state. Nevertheless, as drug administration occurred during both training and testing, Experiment 1 does not permit unambiguous assessment regarding whether the disruption occurred during memory retrieval.

We therefore conducted a follow-up study, in which serotonin levels were manipulated at retrieval. Given our interpretation of Experiment 1, that agonism of the 5-HT1a receptor facilitated temporal averaging (by disrupting selective memory retrieval and/or by directly facilitating an averaging strategy), we hypothesize that blocking the 5-HT1a receptor should have the opposite result, (i.e., it should impair temporal memory averaging). As described in the Section “Introduction,” when rats are trained that different modality cues signal different durations until reinforcement availability (e.g., tone = 10 s, light = 20 s), the presentation of a compound stimulus (tone + light) results in a scalar peak function at an intermediate time (e.g., 16 s), suggesting integration or averaging of the component memories. Because such a procedure results in averaging under non-drug conditions, we deemed that administration of the 5-HT1a agonist would not be able to demonstrate any additional averaging, and would lead to diminished responding to the compound stimulus (for all drug states) due to additional testing under extinction conditions. We therefore elected to examine only the response to a 5-HT1a antagonist (and a saline vehicle as an injection control).

Rats were trained using the PI procedure to time a 10 s duration marked by a tone and a 20 s duration marked by a light, similar to the Tone-short/Light-long group described by Swanton et al. (2009), which showed scalar temporal averaging to the simultaneous compound. We chose to forego counter-balancing the cue-duration relationship in this experiment, as rats trained in the reverse condition (Light-short/Tone-long) initially show scalar temporal averaging (at least with 10 s and 20 s durations), but then progress with continued testing to responding in a skewed manner similar to that seen in Experiment 1 (Swanton et al., 2009). Such responding is difficult to conclusively characterize, as it may reflect a combination of complete and incomplete averaging, selection of different temporal memories on different trials, and/or a bimodal “covering the bases” response strategy.

Once training was completed, rats were given *N*-[2-[4-(2- methoxyphenyl)-1-piperazinyl]ethyl]-*N*-2-pyridinyl-cyclohexan ecarboxamide (WAY-100635), a selective 5-HT1a antagonist, or saline, and tested on their response to the presentation of the non-reinforced compound stimulus (i.e., simultaneous tone + light). Based on the findings in Experiment 1, suggesting increased temporal memory ambiguity and averaging behavior following 8-OH-DPAT administration, we expected that blocking the 5-HT1a receptor would lead to an increase in temporal memory selectivity on the ambiguous compound trials, thereby disrupting the scalar temporal averaging that is consistently seen under these conditions.

### Method

#### Subjects and Apparatus

Ten male Sprague-Dawley rats (Harlan) approximately 90 days of age at the onset of the experiment were subjects. All aspects of housing and the conditioning chambers were identical to that described in Experiment 1.

#### Drugs

WAY-100635 was dissolved in 0.9% physiological saline and administered intraperitoneally immediately prior to half of the test sessions as a fixed proportion of the animals’ body weight (1.0 mg/kg). Drug and vehicle (0.9% saline) injections were counterbalanced across days. Injections began only after all animals completed the training phase of the experiment.

#### Procedure

Rats were trained on a peak-interval procedure to time two different durations (10 s and 20 s) associated with two different discriminative signals (tone and light, respectively). Counter-balancing of the modality-duration relationship was not done for the reasons described above. After stable, temporally controlled responding was obtained to the single cues, the rats were tested on both the trained stimuli and novel compound stimuli (simultaneous tone + light) while under the influence of the drug (WAY-100635) and vehicle. Initial training sessions took place overnight during 12-h sessions. Following acquisition of the criterion durations, training was continued using 2-h daily sessions. Rats were run 7 days a week at approximately the same time each day. Initial overnight training sessions took place during the rats’ dark cycle, while the 2-h training and testing sessions occurred during the rats’ light cycles. All rats were given injections of the vehicle prior to training during the final two training sessions to minimize non-specific effects during testing. The composition of training and testing sessions is listed below.

##### Fixed-interval training (eight overnight sessions)

Each trial began with onset of either a 10 s “short” stimulus (4 kHz, 95 dB, steady tone) or a 20 s “long” stimulus (illumination of the house light). Following the target duration, the first nosepoke in the center aperture terminated the stimulus and a 45 mg grain pellet was delivered to the food magazine. There was no programmed consequence for responding early, and the signals stayed on until a reinforced response occurred. The probability of a tone or light trial was equal (50% each) during fixed-interval training, and trial type for this and all subsequent training/testing sessions was selected at random with replacement. Trials were separated by a uniform variable inter-trial interval of 40–60 s.

##### Peak-interval training (eight overnight sessions, 12 two-hour daytime sessions)

Peak-interval training was identical to fixed interval training, except for the addition of non-reinforced probe trials, which terminated independently of responding following a variable interval that was 3–4 times the “long” criterion duration (i.e., 60–80 s). Reinforcement probability was set such that the reinforcement rate for the two stimuli was equivalent, which is necessary for temporal averaging to occur (Matell and Kurti, 2014). To this end, the number of reinforced tone and reinforced light trials were programmed to be equal, but the number of probe trials differed across cues. Specifically, the ratio of FI to PI trials was 1:3 for the short cue (i.e., 25% reinforced short tone trials), and 1:1 for the long cue (i.e., 50% reinforced long light trials).

##### Testing (10 two-hour daytime sessions)

Testing was identical to peak-interval training, except for the addition of non-reinforced probe trials in which the simultaneous compound stimuli (tone + light) was presented, and rats were given an injection of drug or vehicle immediately prior to each session. Due to the short terminal half-life for WAY-100635 (i.e., 33 min; Zuideveld et al., 2004), compound probe trials were only presented during the first 30 min of each testing session. During this initial period, compound probe trials were randomly selected with 20% probability. Order of administration of WAY-100635 or vehicle was randomized in five, two-day, blocks, such that all rats were tested once following drug administration and once following saline administration within each block.

#### Analysis

Data from the first 30 min of each testing session were pooled for analysis. Given the low numbers of trials and the low response rate following WAY-100635 administration, which hindered assessment of temporal control, responding was binned in 2-s bins. In contrast to Experiment 1, because peak responding was generally symmetrical, pooled response rate functions were fit with a 4-parameter normal distribution, to obtain measures of peak rate (amplitude), peak time (mean), and peak spread (standard deviation). This 4-parameter function did an acceptable job fitting the data, with mean *R*^2^ values of 0.885 (0.133), 0.872 (0.067), and 0.707 (0.196), for the short, compound and long peaks, respectively, on saline sessions and mean *R*^2^ values of 0.800 (0.194), 0.755 (0.166), and 0.460 (0.300) for the short, compound, and long peaks, respectively, on drug sessions. The CV was computed as peak spread/peak time. Single trial analyses were conducted as described in Section “Experiment 1.” Summary data are presented as mean (SD). Repeated measures ANOVAs were conducted with Duration (short, compound, long) and Drug (saline, WAY-100635) as factors. *Post hoc* comparisons were performed as simple effects (i.e., LSD pairwise comparisons) using the overall error term from the omnibus ANOVA. Greenhouse-Geisser correction was utilized if violations of sphericity occurred.

### Results

As a result of a computer malfunction, the rats did not complete the second test session and all data from the session was lost. Rats were consequently given two additional training sessions prior to the resumption of testing. In total, there were a mean of 28.9 (5.4) short probe trials, 9.9 (3.6) long probe trials, and 16.6 (4.8) compound probe trials per rat.

#### Saline Results

**FIGURE 5:**
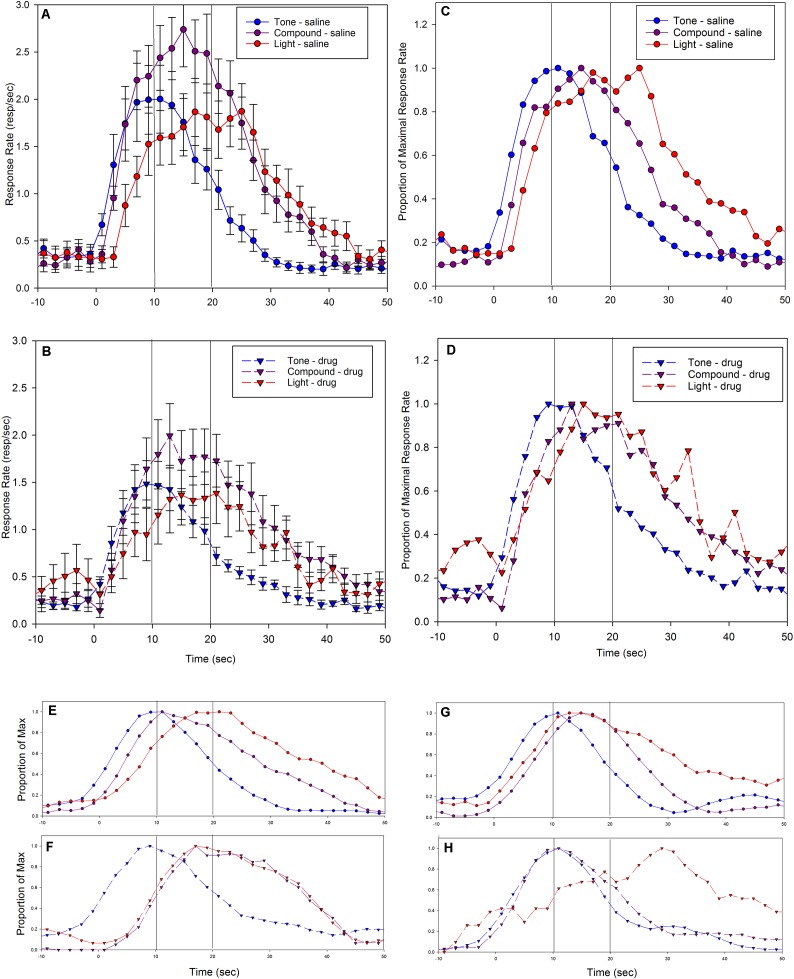
Mean peak response functions from probe trials during test sessions in response to the short (tone) cue = 10 s, the long (light) cue = 20 s, or the compound cue (tone + light). Compound cue trials were never reinforced. Panels **(A,C)** are from vehicle (saline) sessions, and panels **(B,D)** are from drug (5-HT1a antagonist, WAY-100635) sessions. Panels **(A,B)** present raw response rates, as well as indicate standard errors, and panels **(C,D)** present the same data after normalizing by maximal response rates. Panels **(E–H)** depict example peak functions from individual rats. Panels **(E)** (saline) and **(F)** (WAY-100635) show the data from a rat whose compound peak function on antagonist sessions overlapped the long peak function, and panels **(G)** (saline) and **(H)** (WAY-100635) show data from a rat whose compound peak function on antagonist sessions overlapped the short peak function. In all panels, blue indicates tone (short) trials, purple indicates compound (tone + light) trials, red indicates light (long) trials. Circles and solid lines indicate saline data, and triangles and dashed lines indicate drug data. Lines at 10 s and 20 s indicate the programmed time of reinforcement availability.

Mean response rate as a function of time (i.e., peak functions) from the saline sessions are plotted in **Figure 5A**. To allow a better visualization of the temporal pattern of responding independent of the change in response rate, the same data are replotted after being normalized by maximal response rates in **Figure 5C**.

As can be seen, the peak functions for the two component cues were centered near the programmed fixed intervals of 10 s and 20 s, and the long peak function was broader than the short peak function, as to be expected given the scalar property. Specifically, the mean peak time for the short tone cue was 11.5 s (2.2) and it was 20.1 s (4.1) for the long light cue. The mean peak spread was 7.3 s (1.1) for the short cue and 10.5 s (4.7) for the long cue. As a result, relative spread (CV) was 0.65 (0.10) for the short cue and 0.52 (0.19) for the long cue. Additionally, the mean amplitude was 1.9 (1.2) responses per second for the short peak function, and 1.7 (0.9) responses per second for the long peak function.

Presentation of the stimulus compound (tone + light) resulted in a normal shaped peak at a location midway between the two programmed fixed intervals, with a mean peak time of 15.8 s (2.5), and a mean peak spread of 9.1 (1.8), resulting in a CV of 0.58 (0.07). The average amplitude of the compound peak was 2.5 (1.0) responses per second.

#### Drug Results

Mean response rate as a function of time (i.e., peak functions) from sessions following administration of WAY-100635 are plotted in **Figure 5B**, and the same data after being normalized by maximal response rate are replotted in **Figure 5D**. As can be seen, the administration of the serotonergic 1A antagonist appeared to result in a decrease in response rate (**Figures 5A,B** are scaled equivalently), but no change in the temporal control associated with the component cues. The mean peak time for the short tone cue was 12.0 s (2.9) and 20.3 s (5.0) for the long light cue. The mean peak spread was 8.4 s (4.0) for the short cue and 8.5 s (3.9) for the long cue. As a result, the CV (peak spread/peak time) was 0.78 (0.35) for the short cue and 0.44 (0.22) for the long cue.

Presentation of the compound cue again resulted in a peak function that appeared to be of greater amplitude than the component cues. However, in contrast to the compound peak following saline administration, the peak function following WAY-100635 administration appeared to fall closer to the long, light function, nearly overlapping it (**Figure 5D**). The mean peak time of the compound response function fell at 17.8 s (4.6), with a peak spread of 9.2 s (1.9), resulting in a CV of 0.53 (0.10). Inspection of individual subject’s response functions suggested different response patterns to the compound cue in different rats, with compound responding overlapping either the short or long response function. Indeed, the standard deviation (across subjects) of the compound peak time following drug administration was almost twice that of the compound peak time following saline administration.

**FIGURE 6:**
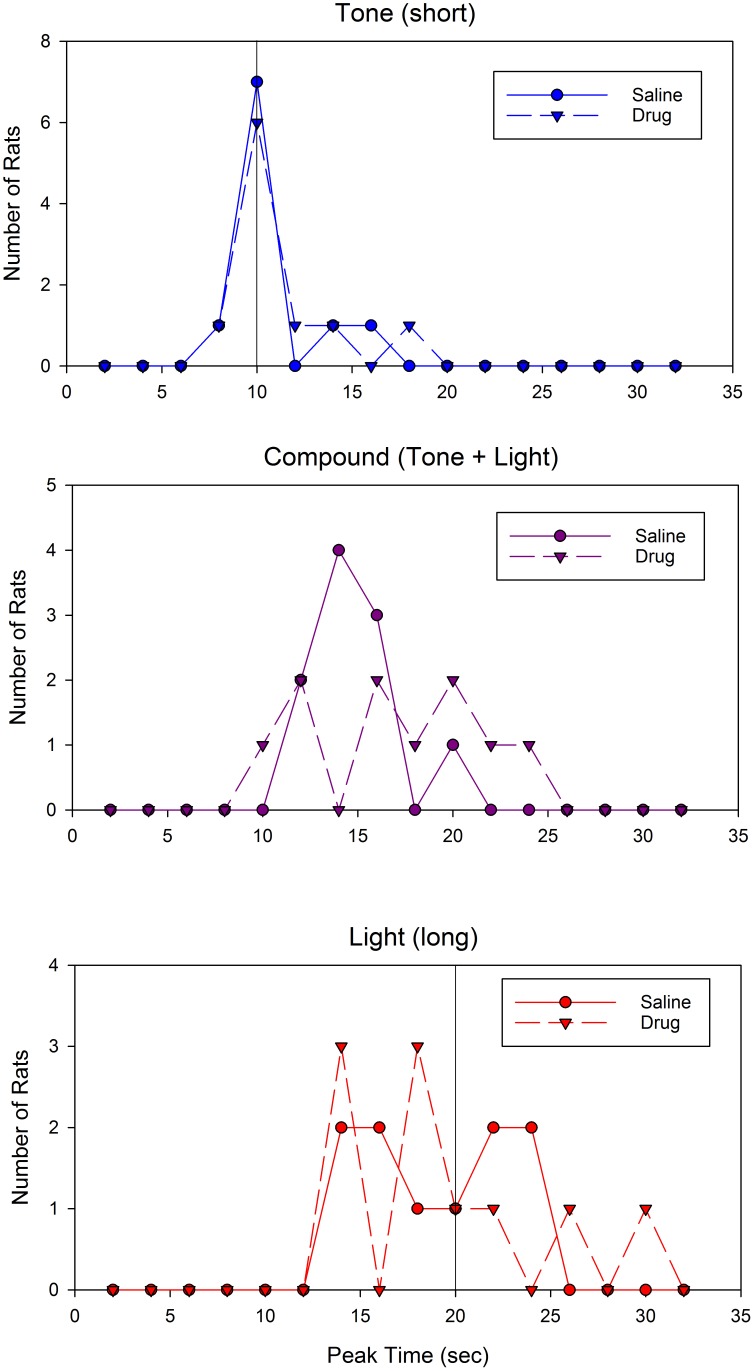
Distribution of peak times following saline or WAY-100635 administration. The **(Top)** panel displays peak times for the short (tone) cue, the **(Middle)** panel displays peak times for the compound (tone + light) cue, and the **(Bottom)** panel displays peak times for the long (light) cue. Colors and symbols match **Figure 5**.

Support for a difference in responding to the compound cue can be seen in **Figures 5E–H**, which show representative individual subjects’ peak functions following saline (**Figures 5E,G**) and drug (**Figures 5F,H**). As can be seen, the compound peak functions fell in between the component peaks following saline administration, but overlap one of the component peaks following WAY-100635 administration. Inspection of the distribution of peak times for the compound cue reveals that administration of the drug led to a flat or perhaps bimodal distribution, whereas the compound peak time distribution following saline administration was clearly unimodal (**Figure 6**, middle). In contrast, the shape of the distributions of peak times for the single tone and light cues did not appear to be impacted by drug administration (**Figure 6**, top and bottom).

The change in the breadth of the compound peak time distribution did not impact the normality of the data as indicated by a non-significant test of normality (Shapiro–Wilk statistic = 0.96, *p*> 0.10). Therefore, we conducted a repeated measures ANOVA with Duration and Drug as within-subject factors. Results demonstrated a main effect of Duration [*F*(2,18) = 36.93, *p*< 0.001], but no effect of Drug [*F*(1,9) = 2.22] and no interaction (*F*< 1). *Post hoc* comparisons across Duration were all significant (all *p*s < 0.05). Similarly, a repeated measures ANOVA on CVs revealed only an effect of Duration [*F*(2,18) = 5.53, *p*< 0.05], with the CV for the short cue being larger than the CV for the compound and long cue (*p*s < 0.05), but no difference between the compound and long CVs (*p*> 0.10). There was no effect of Drug (*F*< 1) and no interaction (*F*< 1). This failure of the scalar property has been seen in our compounding studies before (Swanton et al., 2009; Swanton and Matell, 2011; Matell and Kurti, 2014; De Corte and Matell, 2015), and is likely a result of the lower reinforcement probability for the short cue as compared to the long cue, thereby inducing greater variability in responding (Roberts, 1981; Gharib et al., 2001; Kaiser, 2008; Stahlman et al., 2010). For peak rate, there was a significant effect of Drug [*F*(1,9) = 34.83, *p*< 0.001], as peak rates declined following administration of WAY-100635, and a significant effect of Duration [*F*(2,18) = 6.18, *p*< 0.05], but no interaction (*F*< 1). The effect of Duration was due to the compound peak rate being larger than both the short (*p*< 0.001) and the long (*p*< 0.05) peak rates, whereas these component peak rates did not differ (*p*> 0.10).

We also conducted single trial analyses to identify the times at which the rats abruptly switched from a low rate of responding to a high rate of responding and vice-versa. **Table 2** reports the mean transition times for each cue and drug condition.

**Table 2:** Single trial transition times (Experiment 2).

	Saline			Drug		
	Short	Compound	Long	Short	Compound	Long
Start	9.65	8.76	11.93	10.43	12.11	14.00
*SD*	3.86	2.94	4.52	3.78	3.88	3.81
Stop	23.68	29.29	32.26	24.22	31.67	31.47
*SD*	2.42	3.03	3.86	3.32	5.16	3.48

As expected, the single trial transition times progressed in an orderly fashion, largely bracketing the corresponding peak times. A repeated measures ANOVA with Transition (start, stop), Drug (saline, WAY-100635), and Duration (short, compound, long) as factors was conducted. There was the expected a main effect of Transition [*F*(1,9) = 450.0, *p*< 0.001], the expected main effect of Duration [*F*(2,18) = 19.65, *p*< 0.001], and a main effect of Drug [*F*(1,9) = 9.58, *p*< 0.05], as the transition times were later following drug. The Transition × Duration interaction was also significant [*F*(2,18) = 37.60, *p*< 0.001], with the long start times being different than the short and compound start times, and all stop times being different from one another, except for the compound and long. No other interactions were significant [Transitions × Drug: *F*(1,9) = 2.77, *p*> 0.10], Drug × Duration: *F*< 1, Transition × Drug × Duration: *F*(2,18) = 1.80].

While previous work examining the single trial transitions during compounding demonstrated that the normalized variation in transition times to the compound were no greater than that seen for the component cues (Swanton et al., 2009; Swanton and Matell, 2011), we examined whether that same effect was true here and whether administration of WAY-100635 altered this pattern. Specifically, we computed the CV of the start and stop times within animals (i.e., the standard deviation of these transition times within each subject, normalized by the average transition time for that subject). **Table 3** displays the mean within-rat CVs for the start and stop times.

**Table 3:** Single trial transition CVs (Experiment 2, within subject).

	Saline			Drug		
	Short	Compound	Long	Short	Compound	Long
Start CV	0.73	0.68	0.44	0.73	0.70	0.53
*SD*	0.17	0.18	0.24	0.22	0.21	0.19
Stop CV	0.35	0.26	0.23	0.34	0.29	0.26
*SD*	0.08	0.03	0.12	0.05	0.10	0.12

A repeated measures ANOVA with Transition, Duration, and Drug as factors demonstrated a main effect of Transition [*F*(1,9) = 267.80, *p*< 0.001] as stop times were relatively less variable than start times, as seen previously (Church et al., 1994). There was also a main effect of Duration [*F*(2,18) = 9.36, *p*< 0.005], as the relative variability was largest with the short (tone) cue, and smallest with the long (light) cue. There was no main effect of Drug [*F*(1,9) < 1]. There was also a significant Transition × Duration interaction [*F*(2,18) = 7.25, *p*< 0.005], resulting from the long start time CV being less variable than the short or compound start time CVs. No other interactions were significant (all *F*s < 1). Critically, there was no evidence that the compound CV was larger than the component CVs, suggesting that rats are not switching back and forth between timing the short cue and timing the long cue on different compound cue trials.

**FIGURE 7:**
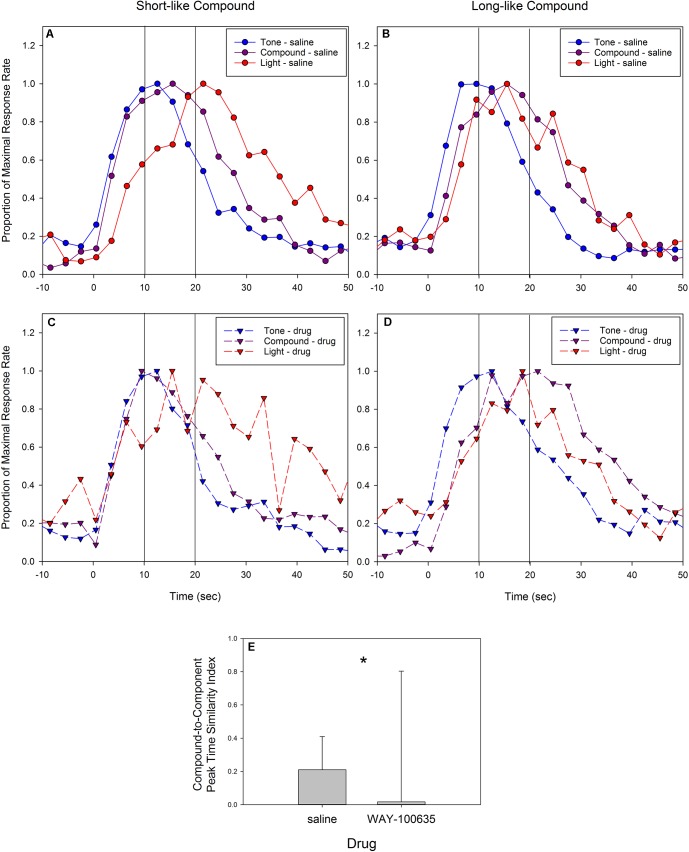
Peak response functions split by whether the compound peak time was closer to the short component cue (tone) peak time **(A,C)** or the long component cue (light) peak time **(B,D)**, based on the compound-to-component peak time similarity index. The top panels **(A,B)** are from sessions following saline administration and the bottom panels **(C,D)** are from sessions following administration of the 5-HT1a antagonist, WAY-100635. Data are plotted after normalizing by maximal response rate. Due to the limited amount of data resulting from splitting subjects by their compound to component cue peak time similarity, response rate data are binned in three second bins for presentation. Panel **(E)** shows the median and interquartile range of the compound-to-component peak time similarity index following saline and WAY-100635 administration. Colors and symbols match **Figure 5**. ^∗^*p*< 0.05.

These data suggest that blocking 5-HT1a receptors lowers the response vigor, but does not impact the temporal control of behavior in response to the trained component cues. However, the overlap between the compound and component cues, and the increase in the breadth of the distribution of compound peak times suggests WAY-100635 may be altering the processes by which rats retrieve multiple temporal memories when presented with the simultaneous component cues. Alternatively, the retrieval processes may operate normally, but the strategies used to deal with the memory discrepancies may have changed. The rats could be switching from an integration-like process to a selection-like process (Matell and Kurti, 2014), and/or changing the relative weights of the two component memories (Matell and Kurti, 2014; De Corte and Matell, 2015, 2016). Differences across individuals in which component memory is selected and/or its weighting, would generate the flatter compound peak time distribution shown in **Figures 6C,D**. To examine this phenomenon, we assessed the similarity between the compound and component peak times. Specifically, we computed a compound-to-component duration similarity index, which identified the relative distance between the compound peak time and the closest component peak times (i.e., the smaller of the following: [(compound-short)/(long-short)] versus [(long-compound)/(long-short)]. If a rat’s compound peak time falls exactly in between its short and long peak time, this similarity index would equal 0.5, whereas if it falls at the same time as one of the component peak times, the similarity index would equal 0. Negative values result if the compound peak time is shorter than the short peak time or longer than the long peak time. Following saline administration, the median compound similarity index was 0.21 (interquartile range = 0.20), whereas following administration of WAY-100635, the median similarity index was 0.02 (IQR = 0.79). These statistics are shown in **Figure 7E**. We note that the error bars (i.e., the inter-quartile range) are quite large following drug administration, and result from the fact that several of the rats’ compound peak functions fell outside the range of their component peak functions, providing further evidence of a 5-HT1a induced disruption of temporal memory processes on compound cue trials. A paired *t*-test demonstrated that the relative distance between the compound peak and the closest component peak was smaller following WAY-100635 than saline [*t*(9) = 2.56, *p*< 0.05]. In contrast, there was no difference in the absolute distance between the short and long peak times following drug administration [*t*(9) = 0.14]. As the scalar property of temporal perception (Gibbon, 1977), as well as the phenomenon of bisection at the geometric mean (Church and Deluty, 1977), indicates that temporal perception may be logarithmic, or that duration similarity is evaluated by ratio comparison, we also computed this similarity index after taking the log of the peak times. Following saline administration, the median similarity index following saline administration was 0.25 (IQR = 0.17), whereas following WAY-100635 administration, the median similarity index was 0.04 (IQR = 0.67). Again, a paired *t*-test indicated that the similarity index became smaller following drug administration [*t*(9) = 2.45, *p*< 0.05], whereas there was no change in the absolute distance between the short and long peak times [*t*(9) = 0.27].

Rats whose compound peak time was closer to the short peak time (using the above similarity index) were classified as having short-like compounds, whereas those whose compound peak time was closer to the long peak time were classified as having long-like compounds. **Figure 7** shows the average peak functions for rats with short-like and long-like compound peaks following saline administration (**Figures 7A,B**) and following drug administration (**Figures 7C,D**).

As can be seen, there was more similarity between the rats’ compound functions and the short or long component functions following drug than following saline. Under saline, five rats had compound peak times more similar to the short peak, and the other five had compound peak times more similar to the long peak. Under WAY-100635, four rats had compound peak times more similar to the short peak, and six rats had compound peak times more similar to the long peak time. Intriguingly, the component peak that the compound cue was most similar to was not consistent between saline and WAY-100635 sessions, as seven of 10 rats switched which peak the compound cue was more heavily weighted toward. Specifically, four of the five rats with short-like compounds following saline had long-like compounds following drug administration, and three of the five rats with long-like compounds following saline had short-like compounds following WAY-100635.

### Discussion

The results from the current experiment suggest that blocking serotonergic transmission at the 5-HT1a receptor does not change temporally controlled behavior in response to single cues (aside from lowering response rates), but it does alter the pattern of responding when multiple cues are presented as a simultaneous compound. Specifically, when presented with a compound cue, rats will typically respond at an intermediate duration with a normally shaped, scalar response form (Swanton et al., 2009; Swanton and Matell, 2011; Kurti et al., 2014; De Corte and Matell, 2015). Indeed, rats given saline prior to testing with the compound cue showed exactly that response pattern in the present experiment. We have interpreted this intermediate, scalar, peak as resulting from a temporal memory integration or averaging process utilized as a strategy for dealing with discrepancy or ambiguity in the expected time of reinforcement. In contrast, when given WAY-100635, the response to the simultaneous compound became inconsistent across subjects, resulting in a flatter distribution of peak times. Computation of the relative distance between the compound and component peak times revealed that WAY-100635 increased the similarity between these values, suggesting responses on compound trials were primarily guided by the memory of a single component duration (i.e., short-tone or long-light) rather than integrating the two component durations.

What mechanism might be responsible for these results? One possibility is that blocking the 5-HT1a receptor may have altered the relative salience of the two component cues. Alternatively, the effect of WAY-100635 may be to alter the averaging or integration process, perhaps by changing the relative weight given to each cue. Previous work from our lab has demonstrated that rats can flexibly weight or bias one temporal memory over the other. This change in the weight given to one cue over the other occurs when one of the cues has greater relative value (Matell and Kurti, 2014), when the rat responds on a duration-specific manipulanda (De Corte and Matell, 2015), or when the two cues have differential reliability (De Corte, 2017).

Although the mechanisms by which this alteration in temporal control occurs cannot be conclusively identified by our data, some evidence supports a change in the relative weighting of the component cues, rather than a change in perceptual salience. First, the peak amplitude was greater in response to the compound cue than it was to either of the component cues following both saline and WAY-100635 administration. If blocking the 5-HT1a receptor resulted in the compound stimulus being perceived as just one of the component cues due to salience differences, this should result in only one of the corresponding temporal memories being chosen, and there should have been no change in peak rate. Secondly, unpublished pilot data from our lab using drug-naïve subjects, in which we examined the response to the compound cue after manipulations to the volume of the auditory cue, suggest that there were no changes in temporal averaging behavior as a result of presumed changes in auditory salience.

## General Discussion

Across two experiments, we found that manipulations of 5HT transmission at the 5-HT1a receptor caused changes in the temporal pattern of responding. When jointly considered, results from these experiments are consistent with alterations in temporal memory retrieval and the response strategy used in the face of temporal ambiguity. In Experiment 1, the administration of a 5-HT1a agonist resulted in peak functions centered at a time between the fixed intervals that the rats were trained on, and which were positively (rightward) skewed as though the rats were influenced by both temporal memories. In other words, the rats’ responses following 8-OH-DPAT suggested increased ambiguity about the expected time of reinforcement. In contrast, in Experiment 2, administration of a 5-HT1a antagonist resulted in a disruption of temporal averaging behavior in response to the compound cue, as though the rats’ temporal expectations became less ambiguous.

While temporal averaging behavior, as inferred from the production of a scalar peak function at an intermediate duration, is often seen under conditions of ambiguity such as stimulus compounding, this response pattern has been found to depend on the component cues having equivalent value (Matell and Kurti, 2014). In contrast, when the cues have different incentive values, the peak function to the compound cue is instead highly skewed, with responding biased toward the more valuable cue. In Experiment 1, the probability of reinforcement for the short and long durations (as signaled by the interoceptive cues) was equivalent. As a result, the rate of reinforcement (i.e., the amount of food earned as a function of the duration of the predicting stimulus) favors the shorter duration cue. Indeed, the anticipated value of signaled reward declines in a hyperbolic manner as a function of delay (Bickel and Marsch, 2001; Mazur, 2001; Green and Myerson, 2004). The greater reinforcement rate of the short cue may therefore account for rats’ tendency to begin responding close to the short duration.

In contrast to the equivalent reinforcement probabilities, and consequential difference in value for the two durations in Experiment 1, in Experiment 2, the cue for the long duration (light) signaled a higher reinforcement probability than the cue for the short duration (tone). Specifically, the probability of the short cue being reinforced after 10 s was 25%, whereas the probability of the long cue being reinforced after 20 s was 50%, with the remaining trials being non-reinforced probes. As a result, the reinforcement rates (at least up to the time of expected reward) was equal (25%/10 s = 50%/20 s). Under these conditions, presentation of the simultaneous compound stimulus (tone+light) results in a unimodal, normally shaped, scalar peak function centered at an intermediate duration, although the precise location of the peak is typically biased toward the longer duration (Swanton and Matell, 2011; Matell and Kurti, 2014; De Corte and Matell, 2015). This bias has been interpreted as resulting from greater weighting of the long duration memory based on the higher reinforcement probability (Matell and Kurti, 2014) or from the resulting increased precision of the temporal expectancy (De Corte and Matell, 2016). In Experiment 2, administration of a 5-HT1a antagonist disrupted this scalar temporal averaging behavior in response to the ambiguous compound cue, and instead led to responding that was strongly biased toward one or the other component durations. While such highly biased, selection-like, behavioral response patterns have been seen before (Matell and Kurti, 2014; De Corte and Matell, 2015), they have occurred under conditions that explicitly favored one of the cues, and individual differences have not been prominent. In contrast, in the current work, the cue and duration that was favored by the rats following administration of WAY-100635 appeared to differ across subjects. It is worth noting that such individual differences in temporal control have been seen previously. For example, Marshall et al. (2014) found that rats who demonstrated better temporal precision on an interval timing task were more likely to choose the delayed option in a delay discounting task than rats with poorer temporal precision. As increased serotonergic transmission has been linked to decreased impulsive choice on delay discounting tasks (Miyazaki et al., 2011, 2014), the present findings and those of Marshall et al. (2014) may indicate that individual differences in serotonergic transmission are an important factor in temporal control and decision making.

Serotonin functioning has been broadly implicated in various aspects of cognition (Glikmann-Johnston et al., 2015; Švob Štrac et al., 2016). However, findings of studies assessing the effects of 5-HT1a ligands tend to be inconsistent and may depend on drug administration dose, route, and task (Leiser et al., 2015). An obvious question is whether the present results showing serotonergic alterations in the response to temporal ambiguity relate to 5-HT1a receptor involvement in interval timing more generally. We found that other than lowering response rate, administration of the antagonist, WAY-100635, had no effect on timing single durations. Conversely, administration of the agonist, 8-OH-DPAT had a differential effect depending on the group-drug assignment (i.e., whether saline = 5 s/agonist = 20 s or agonist = 5 s/saline = 20 s). This bi-directional response is not easily explained by changes in clock speed, which would cause a horizontal shift in single duration timing, nor in clock speed variability, which would cause an increased peak spread in single duration timing. Likewise, changes in temporal memory encoding are inconsistent with the findings of Experiment 2, in that the drug was only administered at test, it only had effects on compound trials, and no reinforcement was given on these trials. Finally, the effects are inconsistent with changes in single duration memory retrieval processes, as changes in single duration sampling would have led to altered response spreads on single duration trials. Thus, the impact of these drugs appears to be selective to processes operating at the time of memory retrieval under conditions of ambiguity. Given that we did not test the impact of a 5-HT1a agonist in Experiment 2, we encourage replication and expansion of our findings through the use of alternative tasks and pharmacological manipulations. For example, it may be the case that administration of 8-OH-DPAT would cause the “incomplete” or “partial” averaging(i.e., non-scalar, positively skewed peak functions) typically seen in light-short/tone-long conditions (Swanton and Matell, 2011) to become “complete” averaging (scalar, symmetric peak functions) as seen in tone-short/light-long conditions (Swanton and Matell, 2011).

These selective effects are consistent with past investigations of serotonin’s role in interval timing. In a review, Ho et al. (2002) concluded that the small and inconsistent effects of 5-HT manipulations on performance in “timing tasks” are primarily mediated by increased response switching behavior. They conclude that serotonergic effects in DRL procedures in which time plays a necessary, but not sufficient, role (O’Donnell et al., 2005), likely result from alterations to non-timing processes, such as the changes in impulsivity, which may also be mediated by the rate of switching between behavioral states. Similarly, changes in inter-temporal choice tasks, which are sensitive to modulation of the serotonergic system (Fonseca et al., 2015; Buhusi et al., 2017; Xu et al., 2017) naturally involve retrieval and comparison of multiple memories of delay to reward, and thus are consistent with this system playing a role in decisions under conflict.

Intriguingly, serotonin has been recently implicated in playing a modulatory role in multisensory integration (Tang and Trussell, 2017). Administration of serotonin led to an increase in the efficacy of the multisensory input, while it decreased the efficacy of single modality input. The current results showing that administration of these same drugs increased (8-OH-DPAT) and decreased (WAY-100635) the utilization of multiple temporal memories suggests that the effects of serotonin may have broad impacts on integrating versus selecting diverse sources of information via 5-HT1a receptors.

To summarize, the studies we described found that administration of serotonergic drugs modulated the use of temporal memories under conditions involving ambiguity. Specifically, use of the 5-HT1a agonist 8-OH-DPAT as a discriminatory cue produced responses influenced by durations learned following saline administration. In contrast, the 5-HT1a antagonist WAY-100635 prevented the complete integration of memories associated with two different cues when these were presented as a compound. Together these results suggest activity of the 5-HT1a receptor plays an important role in retrieval of temporal memories and the resolution of conflicting information.

## Author Contributions

ZS helped to design and conduct the experiments, analyze the data, and write the manuscript. SC helped to design and conduct the experiments and analyze the data. LH helped to conduct the experiments and analyze the data. MM helped to design the experiments, analyze the data, and write the manuscript.

## Conflict of Interest Statement

The authors declare that the research was conducted in the absence of any commercial or financial relationships that could be construed as a potential conflict of interest.
